# The Degree of Mucosa-Associated Microecological Imbalance in Ulcerative Colitis Patients with Different Mayo Score and Its Relationship with Mucosal Mechanical Barrier Damage

**DOI:** 10.5152/tjg.2025.24609

**Published:** 2025-10-09

**Authors:** Yansheng Shang, Xiaohong Wang, Xingyuan Diao, Lixiang Li, Xiuli Zuo

**Affiliations:** 1 Department of Gastroenterology, QILU Hospital of Shandong University, Jinan, China; 2 Department of Gastroenterology, Jinan People’s Hospital, Jinan, China; 3 Department of Gastroenterology, The Affiliated Taian City Central Hospital of Qingdao University, Taian, China

**Keywords:** Bacterial microbiota, fungal microbiota, Illumina MiSeq sequencing, Mayo score, microecological imbalance

## Abstract

**Background/Aims:**

Ulcerative colitis (UC) is a chronic inflammatory bowel disease known for mucosal inflammation and dysbiosis of gut microbiota. The association between mucosa-related microecological imbalance and UC severity is a crucial aspect in unraveling the disease’s pathogenesis. The relationship between mucosa-related microecological imbalance and different levels of UC severity was investigated, as defined by Mayo score, and its association with mucosal mechanical barrier damage.

**Materials and Methods:**

The composition of mucosa-associated bacterial and fungal populations in UC patients and healthy controls was analyzed using Illumina MiSeq sequencing. The analysis focused on changes in the diversity of bacteria and fungi, along with their distribution at phylum and genus levels. Additionally, the potential relationship between microecological imbalance and damage to the mucosal mechanical barrier was assessed.

**Results:**

Patients with severe UC exhibited elevated abundance indexes, increased numbers of phyla, and higher proportions of specific phyla (*Actinobacteria*,* Acidobacteria*,* Chloroflexi*,* Gemmatimonadetes*,* Nitrospirae*, and *an unclassified phylum*) compared to patients with mild or moderate UC. Conversely, the proportions of dominant bacterial phyla (*Firmicutes*,* Bacteroidetes*, and *Proteobacteria*) displayed an inverse relationship with UC severity. In the fungal microbiota, moderate and severe UC cases showed a greater prevalence of negative genera compared to mild cases. Notably, changes in microflora composition were associated with the extent of mucosal mechanical barrier damage.

**Conclusion:**

The progression of mucosa-associated microecological imbalance is associated with increasing inflammation in UC, potentially contributing to disruptions in the intestinal mucosal mechanical barrier.

**Main Points**

- The progression of mucosa-associated microecological imbalance is associated with increasing inflammation in ulcerative colitis (UC).
- Changes in microflora composition were associated with intestinal mucosal mechanical barrier damage.
- The microbial alterations may serve as predictive markers for disease development and severity in UC.

## Introduction

Ulcerative colitis (UC), classified under inflammatory bowel disease, is a chronic inflammatory disease affecting the rectum and colon, with an unknown etiology. The lesions primarily target the mucosal and submucosal layers of the large intestine, presenting clinical symptoms such as abdominal pain, diarrhea, and bloody mucous stools. Notably, the global incidence and prevalence of UC are increasing. The Mayo score, a critical assessment tool in UC management, provides a quantitative measure of disease activity and directs treatment decisions.^1^ While the pathogenesis of UC remains incompletely understood, the intricate interplay between microbial imbalance and UC development is a pivotal research area in elucidating the disease pathogenesis.^2^

The disruption of the microecological balance is a crucial factor in the onset and progression of UC. This imbalance can trigger a cascade of immune responses, facilitating the infiltration of inflammatory cells and enhancing the expression of inflammatory mediators,^3^ consequently exacerbating inflammation in UC. Effectively preventing inflammation in UC serves as a valuable approach for boosting remission rates. Bacteria and fungi stand out as key constituents of the intestinal microbiota, exerting significant effects in this context.^4^^-^^6^ Variations in the bacterial and fungal profiles have been observed in UC patients across different disease stages. For instance, there are significant shifts in bacterial composition between patients with mild, moderate, and severe UC, indicating a progressive increase in microbial dysbiosis with disease severity.^7^ Similarly, fungal communities have been linked to disease activity, with specific fungal taxa correlating with clinical indices, such as the Mayo score.^6^ A classic study revealed noteworthy variances in both bacterial and fungal compositions between UC patients experiencing remission and those in active flare.^4^ Hence, delving into the mucosa-associated microecological imbalance among UC patients at various disease stages holds paramount significance.

The integrity of the mechanical barrier in the intestinal mucosa is essential for regulating physiological functions. This barrier consists of mucosal epithelial cells and their tight interconnections.^8^ In addition, the tight connections of mucosal epithelial cells can directly prevent microbes from crossing the barrier.^9^ Thus, the mechanical barrier plays an important role in maintaining the microbial barrier.^10^ The damage to the mechanical barrier may be related to the degree of microecological imbalance.

Herein, the degree of microecology imbalance in UC patients of different stages at the level of phylum and genus was studied. The Mayo score was used to evaluate the stage of UC.^11^^,^^12^ Meanwhile, the relationship between the microflora imbalance and the damage degree of the mechanical barrier was analyzed. These findings may provide the direction for further research and a more theoretical basis for the prevention and treatment of UC.

## Materials and Methods

### Study Participants

This study was registered at the Protocol Registration and Results System (NCT03151850). All the subjects were recruited from QILU Hospital of Shandong University. They were aged from 18 to 80 years. UC was diagnosed according to the standard clinical, endoscopic, and histological features of UC.^2^ Exclusion criteria: 1) Recent use of certain medications: participants who had taken probiotics, prebiotics, antibiotics, anti-fungal agents, or colon-cleansing regimens for at least 8 weeks before enrollment were excluded to eliminate any potential effects these treatments might have on the gut microbiota composition. 2) Intolerance or other diseases: patients with an intolerance to colonoscopy and those suffering from other gastrointestinal diseases were excluded to maintain focus on UC as the primary condition under investigation. 3) Pregnancy or lactation: female participants who were pregnant or lactating were also excluded, as these conditions can significantly alter microbiota and immune responses. Healthy subjects (HS) who underwent a physical examination during the same period were included as controls. To ensure the integrity of the microbiota comparisons, the following exclusion criteria were applied to the HS group: 1) Recent use of probiotics or prebiotics within 8 weeks before enrollment. 2) Use of proton pump inhibitors or non-steroidal anti-inflammatory drugs. 3) History of gastrointestinal tract surgeries that could affect microbiota and mucosal health. 4) Presence of any gastrointestinal disorders or symptoms suggestive of intestinal conditions, such as abdominal pain, diarrhea, or bloating. This study was performed in accordance with the Declaration of Helsinki and was approved by the Ethics Committee of QILU Hospital of Shandong University (No.: 2016034; Date: 15 August, 2016). All participants have provided written informed consent.

### Patient Grouping

Regarding the grouping of UC patients based on disease severity, According to the Mayo score (a validated tool for assessing UC disease activity),^11^^,^^12^ the participants were categorized into 3 severity levels: mild UC (Mayo score 3-5): Participants who exhibited mild clinical symptoms and minimal endoscopic findings; moderate UC (Mayo score 6-10): Participants with moderate clinical symptoms characterized by increased stool frequency and blood in the stool, along with more pronounced endoscopic findings; and, severe UC (Mayo score 11-12): Participants presenting with significant symptoms, including severe diarrhea, extensive bleeding, and considerable mucosal damage, as assessed through endoscopy.

### Clinical Data Collection

The clinical data of the subjects, including sex, age, the number of diarrhea per day, the degree of hematochezia, mucosa lesions under endoscopy (features, inflammation indexes, the range of inflammation), and pathological features (the changes of goblet cells or gland cells, crypt abscesses, and other severe lesions) were collected.

### Sample Collection

Intestinal mucosa tissues were collected under colonoscopy. Factors that could damage the intestinal mucosa (such as drinking, cold, or spoiled food and drugs) were forbidden before the colonoscopy. The mucosa of typical lesion sites (sigmoid colon and rectum) in the UC group was collected. The mucosa of the sigmoid colon was collected from subjects in the HS group.

### DNA Extraction and Illumina MiSeq Sequencing

Microbial DNA was extracted from all the samples. Then, PCR was performed in a triplicate 20 μL mixture containing 4 μL of 5 × FastPfu Buffer, 2 μL of 2.5 mM dNTPs, 0.8 μL of each primer (5 μM), 0.4 μL of FastPfu Polymerase, and 10 ng of template DNA.^13^ The Sequencing primers to bacterial microbiota were 338F (5’ - ACTCCTACGGGAGGCAGCAG - 3’) and 806R (5’ - GGACTACHVGGGTWTCTAAT - 3’). The primers to fungal microbiota were ITS1F (5’ - CTTGGTCATTTAGAGGAAGTAA - 3’) and ITS2-2043R (5’ - GCTGCGTTCTTCATCGATGC - 3’). Then, the DNA samples were purified using the AxyPrep DNA Gel Extraction Kit (Axygen Biosciences, Union City, CA, USA) and quantified using QuantiFluor™ -ST (Promega, USA). Purified samples were pooled in equimolar and paired-end sequenced (2 × 250) on an Illumina MiSeq platform according to the standard protocols. The raw reads were deposited into the National Center for Biotechnology Information (NCBI) Sequence Read Archive database. The raw fastq files underwent demultiplexing and quality filtering with Quantitative Insights Into Microbial Ecology (QIIME) (version 1.17) based on the following criteria: (i) Truncation of 300 bp reads at sites with an average quality score <20 over a 50 bp sliding window, discarding reads shorter than 50 bp. (ii) Exact barcode matching, allowing a 2-nucleotide mismatch in primer matching, and removal of reads containing ambiguous characters. (iii) Assembly of only sequences with an overlap longer than 10 bp according to their overlap sequence; reads that could not be assembled were excluded. Operational Taxonomic Units (OTUs) were clustered at a 97% similarity cutoff using Universal Parser (UPARSE) (version 7.1) from drive5.com, and chimeric sequences were detected and eliminated with Ultra-fast Chimera Detection (UCHIME). The taxonomic classification of each 16S rRNA gene sequence was conducted using the Ribosomal Database Project (RDP) Classifier against the Silva (SSU115) 16S rRNA database, applying a confidence threshold of 70%.

### Bioinformatics Analysis

The Ace index, Chao index, Sobs index, Shannon index, and Simpson index were used to evaluate the abundance and diversity of mucosa-associated bacterial and fungal microbiota. Next, the compositions and respective proportions of bacterial and fungal microbiota at the phylum and genus levels were analyzed.

### Correlation Analysis of the Mechanical Barrier of Intestinal Mucosa and the Specific Change Rules of Bacterial and Fungal Microbiota

The damaging degrees of the mechanical barrier were estimated from 3 aspects: the depth of the lesion, the scope of the lesion (the extent and anatomical range of mucosal damage), and the degree of damage to the microstructure. According to the clinical information collected above, the depth and scope of the lesion were evaluated using the modified Baron endoscopic grading. The damage degrees of the microstructure were evaluated by histological grading according to the Baron system. At last, the laws of the grading among the 3 groups of UCs were compared with the laws in the bioinformatics analysis.

### Statistical Analysis

The chi-square test, Wilcoxon rank sum test, and Adonis (Anosim) analysis were used for statistical analysis. *P* < .05 was considered significant.

## Results

### Clinical Characteristics of the Participants

A total of 47 participants, including 29 cases in the UC group and 18 cases in the HS group, were enrolled in this study. They were aged 18 to 72 years old. According to the Mayo score, the UC patients were subdivided into 3 groups: 12 cases in the mild UC group, 11 cases in the moderate UC group, and 6 cases in the severe UC group. There was no statistically significant difference in sex distribution between the groups (*P *= .91). The intestinal mucosa tissues of 14 participants (4 cases in the HS group, 5 cases in the mild UC group, 3 cases in the moderate UC group, and 2 cases in the severe UC group) were used for the Illumina MiSeq sequencing of fungi.

### The Coverage and Diversity Indexes of Mucosa-Associated Microbiota

**Figure 1.**
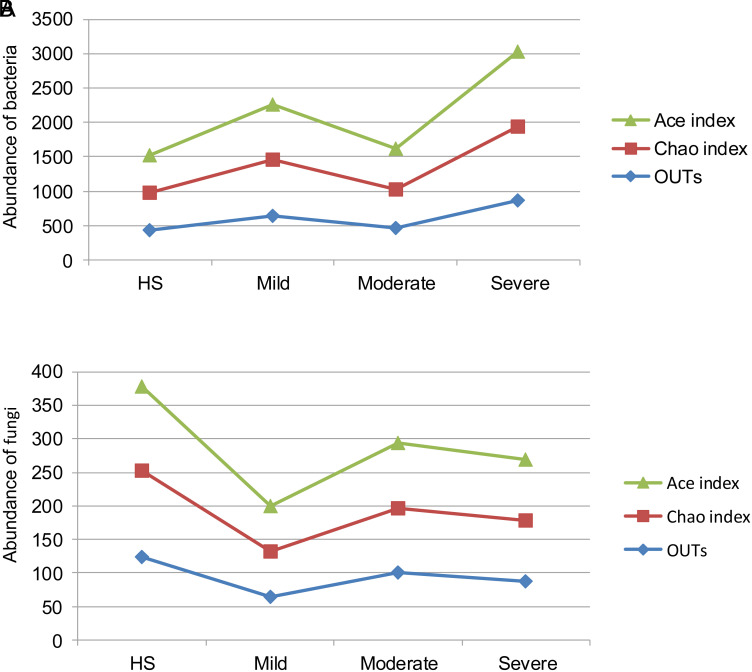
Abundance indexes. (A) The indexes of bacterial microbiota. (B) The indexes to fungal microbiota.

The Illumina MiSeq sequencing showed that the coverage of each group was above 99.20% (99.99%). The abundance of bacteria in UC was higher than that in HS (Figure 1A), with the abundance of fungi showing the opposite case (Figure 1B). The average OTUs of the bacterial and fungal microbiota were 438 (124) in the HS group, 638 (65) in the mild UC group, 458 (100) in the moderate UC group, and 871 (88) in the severe UC group. The average Chao index in HS, mild UC, moderate UC, and severe UC groups was 539.79 (128.33), 820.57 (66.67), 566.67 (96.67), and 1067.33 (90). The average Ace index in HS, mild UC, moderate UC, and severe UC groups was 553.78 (126.67), 805.64 (68.33), 587.52 (96.67), and 1100.13 (91.67). The differences between moderate UC and severe UC in the Chao index (the rank sum test, *P *= .04) and Ace index (the rank sum test, *P *= .03) were statistically significant. Besides, no statistically significant differences were shown.

### The Bacterial and Fungal Micro Dysbiosis at the Phylum Level

**Figure 2.**
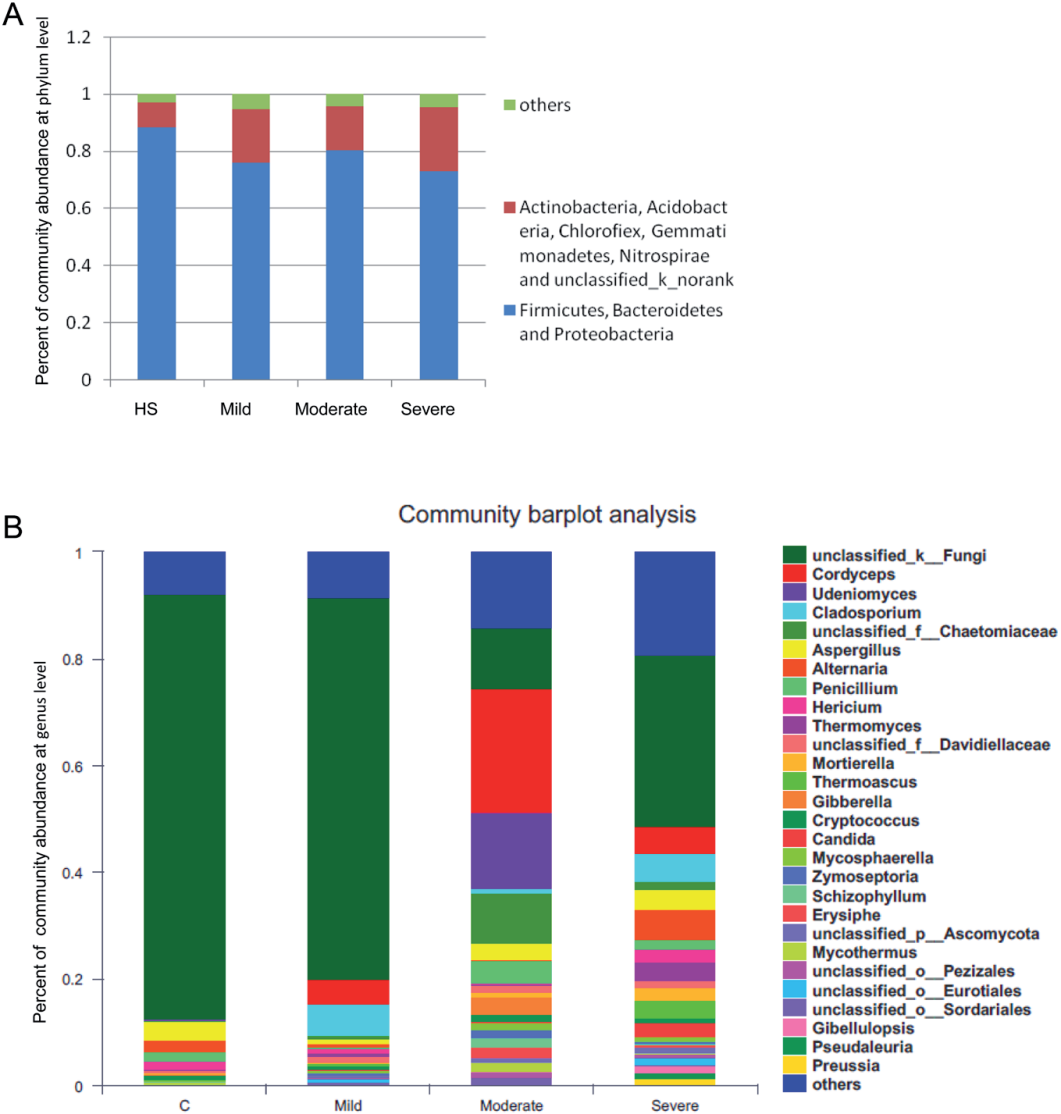
The bacte rial and fungal microecological imbalance at the phylum and genus level. (A) The percentage of the dominant bacterial phyla and the negative bacterial phyla. (B) The percentage of main fungal genera.

The rank sum test showed no statistically significant differences in mucosa-associated bacterial microbiota at the phylum level. *Firmicutes*, *Bacteroidetes*, and *Proteobacteria* were the main phyla of mucosa-associated bacterial microbiota. The percentages of these 3 phyla in HS, mild UC, moderate UC, and severe UC groups were 88.28%, 76.00%, 80.29%, and 73.14%, respectively (Figure 2A). Except for *Proteobacteria*, the types with negative effects included *Actinobacteria*, *Acidobacteria*, *Chlorofiex*, *Gemmatimonadetes*, *Nitrospirae*, and 1 unclassified type (*unclassified_k_noran*k). The percentages of these phyla in HS, mild UC, moderate UC, and severe UC groups were 8.57%, 18.74%, 15.43%, and 22.28%, respectively (Figure 2A). These negative phyla were significantly positively correlated with each group, respectively. The ratio of mild UC, moderate UC, and severe UC was 1:2:4. The average percentage of *Proteobacteria* in the mild group was much higher than that in the other groups, while the percentage of *Actinobacteria* and an unclassified phylum was much higher in the moderate group. In addition, the percentages of the other phyla were much higher in the severe UC group.

*Basidiomycetes* and *Ascomycetes* were the main phyla of mucosa-associated fungal microbiota (>92%). The percentage of *Basidiomycotina* increased in the UC patients while the percentage of *Ascomycetes* decreased. However, no obvious change was observed in other groups.

### The Bacterial and Fungal Microecological Imbalance at the Genus Level

No significant difference was found in the number of dominant bacterial genera and the average proportion of the dominant bacterial genera among the groups. However, the fungal microbiota at the genus level was significant. The main genera in HS, mild, moderate, and severe UC groups were 15, 20, 23, and 26, respectively (Figure 2B). Most of the dominant genera in the UC patients were not detected in the HS group, such as *Cordyceps*, *Candida*, *Preussia*, *Erysiphe*, *Mycosphaerella*, *Cladosporium*, and so on. Furthermore, the average proportion of the dominant fungal genera (an unclassified type) in HS, mild, moderate, and severe UC groups was 79.15%, 70.64%, 11.06%, and 31.49%, respectively (Figure 2B). Rank sum test showed that there were statistically significant differences between mild UC and moderate UC groups (*P *= .03) and between mild UC and severe UC groups (*P *= .04). The percentage of *Candida albicans* in the severe UC group was 2.26% while that in the mild and moderate UC group was far less than 1% (*P *= .01).

### The Correlation Between Bacterial and Fungal Microecological Imbalance and the Damage of Mucosal Mechanical Barrier

The changes in the mucosal mechanical barrier evaluated by the modified Baron endoscopic grading, lesion range grading, and pathological grading were consistent with the changes in bacterial and fungal microecological imbalance (Table 1). In detail, the proportion of grade IV of modified Baron endoscopic grading in mild, moderate, and severe UC groups was 8%, 36%, and 83%, respectively. The proportion of extensive colitis or pancolitis in mild, moderate, and severe UC groups was 0%, 18%, and 100%, respectively. In terms of pathological grading, 16 subjects underwent histopathological examination. Severe lesions included a significant decrease in goblet cells or glandular cells, crypt abscesses, and inflammatory pseudopolyps. The proportion of severe lesions in mild, moderate, and severe UC groups was 12.5%, 50%, and 100%, respectively. The proportion showed an increasing trend, and the proportion in the severe UC group was much higher than that in the non-severe (mild and moderate) UC groups.

Nine subjects who underwent Illumina MiSeq sequencing of fungi further received histopathological examination (Table 2). The proportion of grade IV (extensive colitis or pancolitis, and severe lesions in histopathology) in the mild, moderate, and severe UC groups was 0% (0%, 25%), 50% (50%, 50%), and 50% (100%, 100%), respectively. The proportion showed an increasing trend, and the proportion in non-mild groups was much higher than that in the mild group.

### Anosim Analysis and Adonis Analysis

**Figure 3.**
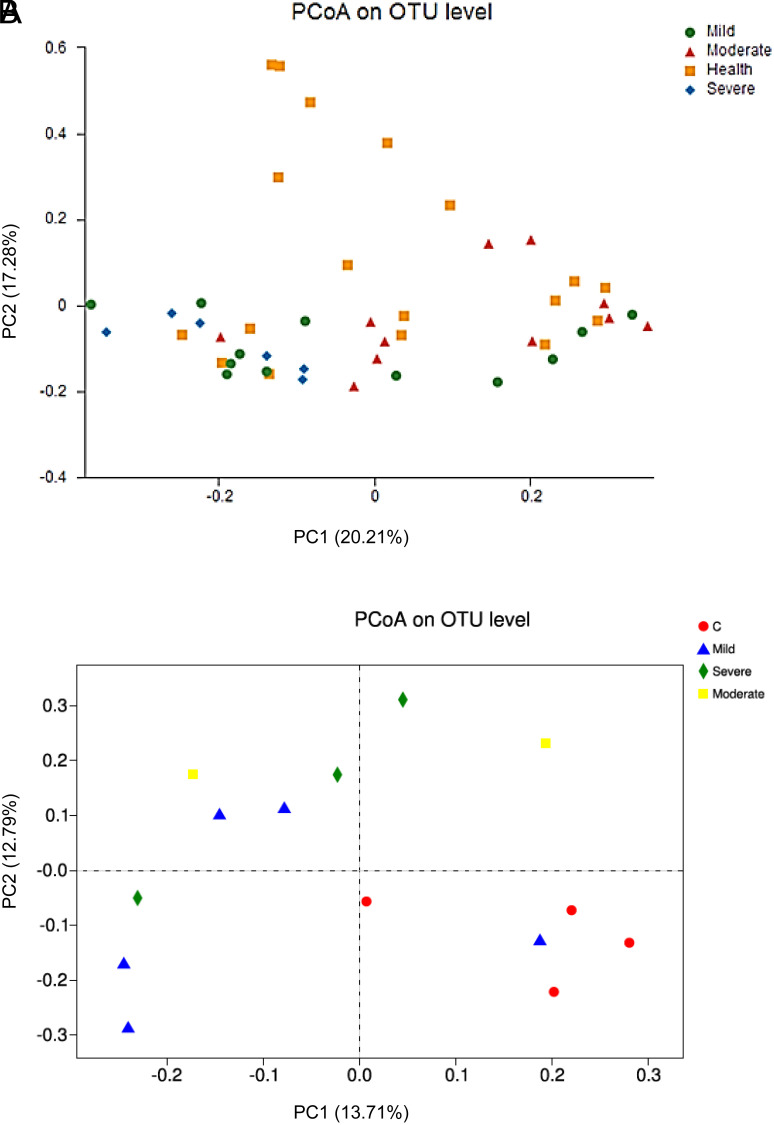
PCoA base d on Unweighted UniFrac distance matrice. (A) The PCoA of the bacterial microbiota. (B) The PCoA of the fungal microbiota.

Based on the Unweighted UniFrac distance matrix, the differences in the mucosal microbiota in the 4 groups were shown by Principal Coordinates Analysis (PCoA) in Figure 3. Statistical differences in the bacterial microbiota between the HS and UC patients were confirmed by Anosim analysis (*P* = .001) and Adonis analysis (*P* = .007) (Figure 3A). Statistical differences in the fungal microbiota between the HS and UC patients were revealed by Anosim analysis (*P* = .028) (Figure 3B). Statistical differences in the bacterial microbiota in the 4 groups were demonstrated by Adonis analysis (*P* = .004). No statistical differences in the fungal microbiota in the 4 groups were shown by Adonis or Anosim analysis.

## Discussion

Previous studies have confirmed the relationship between microecological imbalance and UC.^4^^,^^14^ However, limited studies have explored the correlation between the levels of imbalance and the severity of UC. In this study, the bacterial and fungal composition associated with the mucosa in UC patients at varying stages (based on different Mayo scores) was examined. Although a relatively straightforward procedure, this analysis is more practical for applying and disseminating research outcomes in clinical settings.^11^ The results revealed that all 3 subgroups of UC showed obvious microecological bacterial and fungal imbalance compared with the HS. The bacte rial microecological imbalance of the severe UC group was significantly more serious than that in the non-severe (mild and moderate) UC groups. The fungal microecological imbalance in the non-mild (moderate and severe) UC groups was significantly more serious than that in the mild UC group.

Abundance and diversity are direct indicators of the stability of the intestinal mucosa-associated microbiota,^15^ with changes in these parameters reflecting overall microbial imbalance. This study indicated an increasing trend in the average OTUs and Chao (Ace) index of bacterial microbiota between non-severe UC and severe UC, as well as the average OTUs and Chao (Ace) index of fungal microbiota between mild UC and non-mild UC. The majority of microbiota communities that increased in UC were pathogenic, such as *Chloroflex* and *Candida*.^16^^,^^17^ These results confirm that the colonization and proliferation of negative communities may be the direct factors for the increase of abundance indexes.^7^ Consequently, elevated abundance indexes signify a more pronounced microbial imbalance.

Phylum is the primary classification unit of the microbial community, while genus is the basic unit of the microbial community. The changes in bacterial and fungal microflora at these 2 levels also sufficiently reflect the relationship between the degree of imbalance and the degree of inflammation. Compared with non-severe UC, the percentage of the 3 phyla (*Firmicutes*, *Bacteroidetes,* and *Proteobacteria*) decreased dramatically in severe UC, indicating that the stability of the microflora shows a decreasing trend. Abundant studies have confirmed that *Firmicutes*, *Bacteroidetes,* and *Proteobacteria* are the dominant bacterial microflora phyla.^2^^,^^4^ Similarly, the decrease in the dominant genus of fungal microflora (an unclassified type) in non-mild UC also showed a decreasing trend of microflora stability. However, more studies are needed to explore this unclassified fungal genus. One previous study reported that the ratio of *Ascomycota* to *Basidiomycota* was considered to be positively related to UC.^4^ However, the relationship between the ratio and the Mayo score was identified in the present study.

Additionally, the results indicated that the function of the microecological barrier showed a decreasing trend. Firstly, the percentage of the phyla (*Actinobacteria*, *Acidobacteria*, *Chlorofiex*, *Gemmatimonadetes*, *Nitrospirae,* and *one unclassified type*) increased dramatically in severe UC. The association between *Actinobacteria* and UC has been established,^18^ while* Chloroflex* has been linked to colon cancer.^16^ Secondly, the number of fungal genera with negative effects gradually increased with the development of inflammation. Among these genera, the change of *Candida* was widely consistent with other reports.^19^^,^^20^ It has been recognized as a pathogenic bacterium and can aggregate intestinal inflammation in combination with dextran sulfate sodium.^21^ Different from the previous study,^22^ *Aspergillus* did not show a marked difference between the HS and the UC.

Next, the possible mechanism of the microecological imbalance was analyzed. The destruction of the mechanical barriers may be the fundamental reason.^23^ ^24^ While the Montreal and Paris classification systems are acknowledged for anatomical localization of inflammation, the modified Baron score was employed in this study, as it effectively reflects the relationship between the anatomical scope of inflammation, histopathological severity, and the degree of microbial dysbiosis in UC patients. These results demonstrated that the depth of the lesion, the scope of the lesion (the extent and anatomical range of mucosal damage), and the degree of microstructure damage were positively related to the degrees of UC and the imbalance of the mucosa-associated microflora. It has been reported that mucosal barrier damage is the primary event in inflammatory bowel disease.^23^ Moreover, the mucous layer, an important part of the intestinal mechanical barrier, is difficult to be colonized by pathogenic bacteria in HS or UC patients in remission.^10^^,^^25^ However, the mucous layer of UC patients in the inflammatory period is thin and could be colonized easily by pathogenic bacteria.^25^ Besides, the loss of goblet cells has also been linked to changes in microbial diversity.^10^ It is shown that the number of goblet cells and the microbial diversity changed more significantly in the inflammatory phase compared to the remission phase.^10^ Even more interestingly, a similar correlation was shown from the abundance index, the percentage of the main phylum (genus), and the number of disease-causing phylum (genus). These findings demonstrate that the more seriously the barrier is damaged, the more obvious the imbalance is.

However, the changes in bacterial and fungal microbiota in UC patients with different stages were not consistent with each other. The imbalance of bacterial microecology in severe UC was more serious, while the imbalance of fungal microecology in non-mild UC was more serious. The most likely reason is that the number of participants involved in fungal sequencing was small, which affected the results. Further studies with larger sample sizes are needed to verify this conclusion.

This study has some limitations. First, the sample size for fungal sequencing is limited (only 14 participants). In the future, the plan is to recruit additional participants for fungal sequencing to improve the statistical power and reliability of the findings. Second, there is an unbalanced distribution across mild, moderate, and severe UC groups, which may introduce potential bias. In future studies, a more balanced recruitment strategy will be used to ensure equitable representation.

In summary, the findings underscored a progressive increase in mucosa-related microecological imbalance with more severe UC, characterized by alterations in the composition and diversity of both bacterial and fungal populations. Notably, the study identified specific dysbiosis patterns associated with UC severity, such as elevated abundance indexes, changes in phyla prevalence, and shifts in dominant bacterial and fungal genera. While these microbial changes may have potential as indicators of disease activity, it is important to emphasize that their identification requires invasive procedures such as colonoscopy and biopsy. Furthermore, the results emphasized a correlation between the extent of mucosal barrier damage and the degree of microecological imbalance, indicating that disruptions in the mechanical barrier may contribute to the progression of inflammation in UC. However, establishing a definitive causal relationship between microbial dysbiosis and UC remains challenging. The current findings suggest that microbial dysbiosis may arise as a consequence of UC, with the disease potentially leading to alterations in microbial composition. Simultaneously, microbial imbalances could contribute to the onset and progression of UC, possibly by inducing immune dysregulation and impairing the intestinal barrier function, which may exacerbate inflammation. Future studies are warranted to validate these findings, elucidate these dynamics, and assess the potential role of microbial alterations as predictive markers for disease severity and activity in UC.

## Figures and Tables

**Table 1.** The Features of Modified Baron Endoscopic Grading, Lesion Range Grading, and Pathological Grading in All of the Participants

Group	Mild UC	Moderate UC	Severe UC
Grade IV of endoscopic grading	1	4	5
Grade I-III of endoscopic grading	11	7	1
Proctitis or proctosigmoiditis	12	9	0
Extensive colitis or pancolitis	0	2	6
Severe histopathological lesions	1	3	3
Non-severe histopathological lesions	7	6	0

UC, ulcerative colitis.

**Table 2.** The Features of Modified Baron Endoscopic Grading, Lesion Range Grading, and Pathological Grading in the Participants Who Participated in the Illumina MiSeq Sequencing of Fungal Microbiota

Group	Mild UC	Moderate UC	Severe UC
Grade IV of endoscopic grading	0	0	1
Grade I-III of endoscopic grading	5	2	1
Proctitis or proctosigmoiditis	1	1	0
Extensive colitis or pancolitis	0	1	3
Severe histopathological lesions	1	1	3
Non-severe histopathological lesions	3	1	0

UC, ulcerative colitis.

## Data Availability

The data used for the analyses are available upon reasonable request from the corresponding authors.
